# Address

**Published:** 1877-01

**Authors:** 


### ADDRESS.

*Delivered before the Ohio State Dental Society, by the retiring President, Dr. C. R. Taft, Dec. 7th, 1876.*

GENTLEMEN OF THE OHIO STATE DENTAL SOCIETY: Although I find nothing in your Constitution or By-laws making it compulsory on the part of your retiring president to deliver an address on vacating his office, yet recognizing the fact that custom makes rules quite as arbitrary as fixed laws, I willingly follow in the footsteps of my predecessors, and all the more so as it affords me the opportunity of expressing to you the profound sense of gratitude I feel for the honor you conferred upon me in selecting me as your presiding officer. In the short space of time alloted to an address of this kind, I have thought best to invite your attention to the practical in our profession rather than the theoretical. I do not propose to examine the building up of the tissue, the nature of a cell or molecule, or penetrate to that deeper hidden mystery, the origin of life which is just now engaging the attention of a school of scientists known as evolutionists, represented by such great lights as Darwin, Huxley, Tyndall and Hæckel, for I take it for granted gentlemen it matters little to you (other than it might gratify your curiosity) to know whether it was just four hundred million of years or two hundred millions of years since this busy world of ours cooled down sufficiently to admit of animal life, and whether of this inconceivable space of time it took fifty or one hundred millions of years to develope the sagacious little monkey from which you all sprung. These men are trying to find out what life is. They are praying for some knowledge that will enable them to bridge over the immense chasm between the living and the not living. They have traced life down and down until they have found a speck of glue like, transparent, colorless matter called a bioplast, in the bioplasmic mass they find life and organization but life preceding and he cause of organization. What is this wonderful life force within bioplasm rendering it capable alike of weaving a nerve tendon or muscle? Our scientists thus far have only named it a "stimulus" better far have said God, and their immeasurable chasm is spanned. There is a limit to human knowledge as well as human power. We may know how the rain drops are formed, but we can not call down the gentle showers that slake the thirst of the parched earth, nor stay the mighty thunderbolt. In trying to solve the mysteries of the origin of life, these profound thinkers have penetrated to a barrier which they can neither get over, through, nor around. Better far adopt the older and satisfactory explanation, "God created man in his own image, in the image of God created he him." But what of this am I commencing an argument against scientific research by members in our profession, not at all, I bid all so inclined, God speed, and thank them for all they have given and are giving us of truth. But if we can not all become great scientists, we can at least receive enough of reflected light to become practical useful members of our profession and do much towards alleviating the ills of our suffering patients. With the number of our dental colleges, the excellence of our standard and periodical literature, he must be indolent indeed who has not acquired sufficient knowledge to enable him to fulfill the requirements of his daily routine practice. While we claim to be, and are entitled to, recognition as a profession, we are artisans and should be artists as well. We are some of us anxious for recognition by the medical profession, as if we needed some of the glory of that learned body to help us along. I do not think we should be greatly disturbed by want of it.

"Honor and shame from no condition rise,
Act Well your part there the honor lies."

We should be quite willing to stand squarely upon our merits and in the end we will probably receive all the recognition, honor and praise we are entitled to whether as scientists, artists or artisans. This is an age of new theories, new modes of practice, and new inventions. We are receiving much that is true and good, but I need hardly recall to your minds how many of the theories pertaining to our profession which were maintained with the greatest positiveness only a few years ago, have been abandoned as false and have given way to other newer and more attractive ones, many of which in their turn will be blown away by a breath of truth or be supplanted by others still newer and more plausible.

We were taught, and used to believe that the sac at the point of the root of the devitalized tooth secreted the pus discharged from it, to-day we are told it has nothing to do with it. Are we to infer that this coagulated lymph remains there just to see it well done? Neither the old theory nor the new has left within our reach any practical sure remedy for the difficulty and we have our little abscess to trouble us all the same. New theories are presented to us almost daily, but in adopting them, what we seem most to want, is that we can have thoroughly practical beneficial results growing out of them.

It is scarcely of more consequence to us that we should know the exact nature of the substance contained in the tubuli of dentine than we should have some sure means of lessening the torture inflicted in excavating a sensitive tooth.

We think we understand pretty well the nature of the agents that produce the different varieties of decay which eat away the tooth substance and exposes the delicate pulp, but do we all understand equally well how to cover over this sensitive member so that we may have a living, healthy, serviceable tooth?

We are too apt to adopt hobbies in our profession. Having gotten through with that horrible practice (if we ever adopted it) of eradicating and preventing decay by cutting away the teeth until they looked like so many sharpened pegs or saw teeth and giving the unfortunate victim of such practice the appearance of belonging to the strictly carnivorous class of animals. We are ready for the next new thing and we have it in a revival of the idea most extensively practiced, though not originated by Hunter, of replanting and transplanting teeth.

The advocates of this practice seem to possess all the zeal and enthusiasm usual to new converts. An eminent practitioner has expressed it as his belief that a tooth could be extracted and carried in the pocket for six months and afterwards replanted and do well. It has become the custom with some when the diseased tooth does not yield readily to treatment to extract it, fill and replant, and they tell us their troubles are at an end. It will be well perhaps to go cautiously in the practice of replanting teeth. We only hope that its advocates may secure better and more lasting results than were attained by Hunter, for we have it recorded of him by those who followed him and carefully observed the results of his operations, that had he known the final results of his practice he never would have advocated it.

We are not advised that those who have revived this practice claim to have received any new light or that their practice differs from his, other than that they carefully scrape off every particle of the investing membrane surrounding the root, so that the living membrane within the sockets may have nothing to unite to. One thing we seem to want most just now, is greater uniformity in our modes of operating (for we can not all be doing the best, while we have a dozen or twenty different ways of doing the same thing) it is true that the few, those who are considerd our finest operators do work much alike, but taking the profession as a whole and judging by what we see in our daily practice, are we all working up to the knowledge we possess? That we have made great advance and are progressing rapidly in the matter of filling teeth no one will question, still we need more thorough work, greater care in the preparation of teeth for filling, as well as a nicer adjustment of gold to the borders of the cavities, and especially should we dress down and finish our fillings better than many of us do.

The wonder is that with the well understood welding properties of cohesive gold, the facility with which it can be used as a filling for the teeth. The number and excellence of appliances at hand; that we should find any really poor filling; that we do find so many is attributable I think to a want of care and nice manipulative ability. It is with pride I call attention to the fact that we have here in our own State Society, among you gentlemen some of the finest operators this country or the world has produced. We should all aim to follow the high examples they are giving us. We all admit that the operative branch stands first and foremost in our profession, and find abundant evidence of this in the fact that to this almost exclusively has been given the best energies of many of the best men in the profession, and has it not cost us something in another direction?

Does the mechanical branch stand where we would like to see it? Or where it did twenty years ago? Is the skill displayed or the results attained better than they were then? The almost entire absence of the beautiful specimens of gold plate work we then saw, and the abundance of sets of teeth mounted on rubber without regard to beauty, hideous in the expression they give to the features of the wearer, illy fitted, and worse finished. All would seem to say we have made little progress, but rather retrograded. Can we afford to ignore what we choose to call the mechanical part of our profession? Shall we degrade the mechanical while we exalt the operative? I say no, for I regard it a nice distinction which makes it a greater honor to make a beautiful filling in your patient's mouth, (a purely mechanical operation) or make an equally fine artificial denture at your bench. We may ignore the mechanical, if we will, but it is hardly to our credit that we are allowing it to drift into the kind of hands we are. We may educate and educate the people to a care of their natural teeth as we will, and yet we can not doubt that teeth in great numbers will be lost, and their places will have to be supplied by artificial substitutes. We can no more expect the physician to so instruct his patients in the laws of health that they will no longer require his physic, than we can expect to teach ours to save their teeth, hence we should recommend and be able to supply to our unfortunate patients the very best in the way of artificial substitutes. While we are gaining in one direction we should be careful not to lose in another. We should investigate and learn all and everything likely to prove of advantage to us, and we should at the same time keep our practice fully abreast with our scientific attainments.

Let us seek for and learn well how to use all of those medicinal agents and appliances that will enable us to give to our patients the best and most satisfactory practical results, feeling they will give us as much credit for what we do, as for what we might know, and as it is probably given to few of us to know all of the knowable we should confine ourselves to those studies and adopt such modes of practice as will enable us to do the greatest amount of good to the greatest number of our suffering fellow creatures.

